# Physical activity, sedentary behavior, and adolescent health: a narrative review

**DOI:** 10.3389/fpubh.2026.1809745

**Published:** 2026-04-23

**Authors:** Cong Kang, Liqiang Li, Xianzhong Huang, Feng Jia

**Affiliations:** 1 Department of Physical Education Teaching Research, Shangluo University, Shangluo, Shaanxi, China; 2 School of Physical Education, Xizang Minzu University, Xianyang, Shaanxi, China; 3 Qinling Institute of Biological Resources, Shangluo, Shaanxi, China

**Keywords:** adolescent physical activity, exercise intensity, mental health, narrative review, physical health, sedentary behavior

## Abstract

**Background:**

Participation in exercise during teenage years is vital for shaping physical health and mental wellbeing, which in turn impacts health outcomes later in life. This review seeks to compile and assess the current research regarding the links between physical activity, inactivity, and the health of adolescents, emphasizing both mental and physical effects.

**Methods:**

A focused exploration of various databases, including EBSCOhost, ISI Web of Science, MEDLINE, PubMed, ScienceDirect, and Scopus, was performed for research articles released between November 2014 and January 2026. A deliberate choice of significant observational, interventional, and mechanistic studies was made to create a thorough summary. The integration utilized a narrative method to examine the subtleties of intensity, category, context, and the fundamental physiological and psychosocial mechanisms.

**Results:**

Findings suggest that engaging in moderate to vigorous physical activity greatly enhances both the physical and mental wellbeing of adolescents, with high-intensity workouts being particularly effective for alleviating stress. Extended periods of sitting and excessive use of screens independently heighten the likelihood of obesity, mental health issues, and musculoskeletal discomfort. These associations are notably influenced by factors such as gender, cultural context, and socioeconomic status.

**Conclusion:**

This analysis highlights the intricate nature, progress, and persistent obstacles in this field. To promote the best health outcomes for young people, it is essential to adopt a comprehensive and context-aware strategy based on solid evidence, which enhances the links between research, real-world practices, and policy formulation. By improving research methods, investigating fundamental processes, and developing customized approaches, we can more effectively enhance the wellbeing of adolescents and tackle increasing issues associated with chronic diseases and mental health.

## Introduction

Globally, less than 20% of adolescents meet the physical activity guidelines established by the World Health Organization (WHO). Insufficient physical activity is linked to heightened health risks during adolescence and into adulthood, including conditions such as diabetes, hypertension, obesity, and mental health disorders (1). Furthermore, participation in physical activities during teenage years is vital for fostering healthy habits and promoting metabolic health in the future (2). Given the escalating prevalence of sedentary lifestyles among young people, understanding the multifaceted relationships between physical activity, sedentary behavior, and adolescent health has become a public health priority.

The mental wellbeing of adolescents is a significant global issue, with rising rates of anxiety and depression. This phase of development is crucial for early intervention, as mental health challenges that arise during these years often continue into later life. An increasing amount of research highlights the positive impact of moderate to vigorous physical activity (MVPA) on the mental health of youth, with some findings indicating that even short bouts of 15 min can produce favorable outcomes (3). The advantages for mental health go beyond merely alleviating symptoms, also fostering improved self-esteem, life satisfaction, and cognitive abilities. Nevertheless, the precise mechanisms behind these relationships are not fully understood, and there are ongoing inquiries into the ideal intensities, types, and settings of physical activity that provide the greatest mental health benefits.

Evidence consistently indicates that engaging in regular physical activity leads to better body composition, enhanced cardiorespiratory fitness, and a lower likelihood of chronic illnesses. For instance, studies on Korean adolescents revealed that certain levels of activity—especially exercising three to four times a week or five or more times a week for girls—were linked to obesity risk, underscoring the need to encourage strength-training and minimize sedentary behavior (4). Fitness programs that combine educational resources and behavioral strategies have proven to significantly enhance the health-related quality of life for overweight teens, positively affecting their physical fitness, emotional health, social connections, and academic performance (5). Additionally, recent research using device-based tracking has shown that even light physical activity can provide cardiovascular advantages, while more intense exercise is more strongly correlated with improvements in cardiorespiratory fitness and stress relief (6, 7).

Regardless of how much physical activity one engages in, prolonged sedentary behavior—especially excessive time spent in front of screens—has been identified as a separate risk factor affecting the health of adolescents. Extended periods of sitting and screen use correlate with higher chances of obesity, musculoskeletal discomfort (notably in the lower back and neck/shoulder areas), and mental health issues. Notably, these associations remain significant even when considering levels of physical activity, indicating that sedentary behavior have their own distinct impact on health outcomes. Recent research has focused on the relationship between physical activity, sedentary time, and sleep within the framework of a 24-h movement pattern. Studies employing isotemporal substitution have shown that shifting time away from sedentary activities towards physical exercise or sleep can lead to considerable health improvements (3, 8).

Despite extensive research exploring the associations between physical activity, sedentary behavior, and adolescent health outcomes, critical gaps persist in the existing evidence base. First, while moderate-to-vigorous physical activity (MVPA) has been well-documented in existing literature, the independent health effects of light-intensity physical activity (LPA), as well as the potential health benefits of replacing sedentary time with LPA, have not been sufficiently synthesized. This gap constrains our understanding of the full spectrum of movement behaviors that support adolescent health (2, 6, 9). Second, sedentary behavior (particularly screen time) has been established as an independent risk factor for adverse health outcomes, yet the heterogeneous health impacts of distinct sedentary activity subtypes (e.g., passive television viewing vs. interactive screen use) and their co-occurrence with other unhealthy behaviors such as insufficient sleep remain understudied (8, 10–12). Third, although evidence confirms that the intensity, type, context, and timing of physical activity may have divergent effects on health outcomes, these nuances have not been systematically integrated into a unified analytical framework. Fourth, the psychosocial pathways linking physical activity to mental health (including mediating factors such as self-esteem, depressive symptoms, life satisfaction, and self-rated health) have been evaluated in discrete individual studies, but there is a lack of comprehensive synthesis of these mechanisms across different activity frequencies and intensity levels (13, 14). Fifth, intervention studies have verified the potential of school-based programs, digital health tools, and culturally adapted approaches to improve adolescent health, yet questions remain regarding the optimal design features, target populations, and contextual moderators that can maximize intervention effectiveness (15, 16).

Addressing these gaps is a critical prerequisite for developing evidence-based, context-specific strategies to promote adolescent health.

Considering the broad range, swift progress, and ongoing ambiguities in this area, a narrative review that consolidates existing evidence while thoughtfully analyzing methodological concerns and theoretical frameworks is both relevant and essential. In contrast to systematic reviews that focus on specific inquiries through structured methodologies, narrative reviews facilitate the incorporation of various types of evidence, investigation of fundamental processes, and recognition of key themes and obstacles (17, 18). This method promotes a more adaptable and interpretative interaction with the literature, encouraging deeper discussions regarding the implications for research, practice, and policy.

This narrative review seeks to: (1) consolidate existing research on the links between physical activity, sedentary behavior, and mental as well as physical health outcomes in young people; (2) investigate how factors such as the intensity, type, context, and timing of activities influence these connections; (3) delve into the psychological and physiological processes that could explain the observed relationships; (4) assess the methodological issues and constraints present in the current body of evidence; and (5) highlight key areas for future research and their implications for health promotion and policy formulation.

This review defines physical activity (PA) as any movement of the body that is generated by skeletal muscles and leads to energy expenditure. The intensity of these activities is categorized by metabolic equivalents (METs) as follows: sedentary behavior (SB, less than 1.5 METs), light physical activity (LPA, 1.5–2.9 METs), moderate physical activity (MPA, 3.0–5.9 METs), vigorous physical activity (VPA, 6.0–8.9 METs), and very vigorous physical activity (≥9.0 METs). Activities classified as moderate-to-vigorous physical activity (MVPA) are those that reach or exceed 3.0 METs. Additionally, screen time refers to the duration spent engaging with electronic screens for purposes such as entertainment, communication, or educational activities.

## Methods

### Information sources and search strategy

This piece presents a narrative review that utilized a targeted search strategy to identify significant and impactful evidence for developing a thorough analytical framework, rather than performing a complete systematic review (1). To improve the clarity of the search and screening methodology, a PRISMA-style flowchart is included, though it does not suggest full compliance with the entire PRISMA systematic review guidelines. We searched multiple databases, including EBSCOhost, ISI Web of Science, MEDLINE (PubMed), ScienceDirect, and Scopus, concentrating on research published between November 2014 and January 2026. The aim was to investigate the relationships between habitual physical activity levels and sedentary behavior, as well as their impacts on both physical and mental wellbeing in young people. A mix of thesaurus and free-text keywords was used, addressing topics such as physical activity (e.g., exercise), psychological effects (e.g., mental health, cognitive abilities), sedentary behavior, age groups (e.g., adolescents, young adults), and types of publications.

### Search and inclusion criteria

A screening procedure consisting of two distinct phases was utilized for selecting studies. Initially, two separate reviewers evaluated the titles and abstracts based on established criteria for inclusion and exclusion. Following this, the complete texts of articles deemed potentially suitable were obtained and examined to make a final determination. Any differences in opinion between the reviewers were settled through agreement or, if required, by involving a third reviewer.

To be included in this review, studies had to fulfill specific criteria: (1) Participants were adolescents between the ages of 10 and 19, aligning with the World Health Organization's definition of adolescence. This age bracket was selected as it signifies a developmental stage marked by significant physical, psychological, and social transformations, which differ from those experienced in childhood and adulthood; (2) The research needed to explore the relationship between at least one dimension of physical activity and one or more mental health indicators (such as depression, anxiety, or self-esteem), physical health metrics (like obesity, fitness levels, or chronic illnesses), or cognitive abilities; (3) The focus of the study had to be on physical activity, sedentary behavior, or screen usage; (4) The work had to be an original peer-reviewed publication (including observational and intervention studies) or a systematic review/meta-analysis. Preschool-aged children were excluded due to their unique environmental and social circumstances compared to older children. Only articles published in English and available in full text were taken into account.

### Data charting, analysis, and synthesis

CK handled the charting process, while QLL managed the review phase. The research team pinpointed and expressed essential features of the studies included, summarizing these during the data charting and synthesis phases. When articles lacked vital information, additional online searches were conducted to clarify the links between physical activity, sedentary behavior, and adolescent health. In cases where multiple articles covered the same subject, the one with the most detailed information was chosen for citation, although all articles and references listed in the charts were recorded. Each article was scrutinized, and the charting information was discussed by at least two researchers, revised, and re-evaluated until a consensus was reached. A narrative synthesis method was utilized to meet the review goals, focusing on identifying, describing, and evaluating the relationships within the research topic.

## Results

### Search results

**Figure 1:**
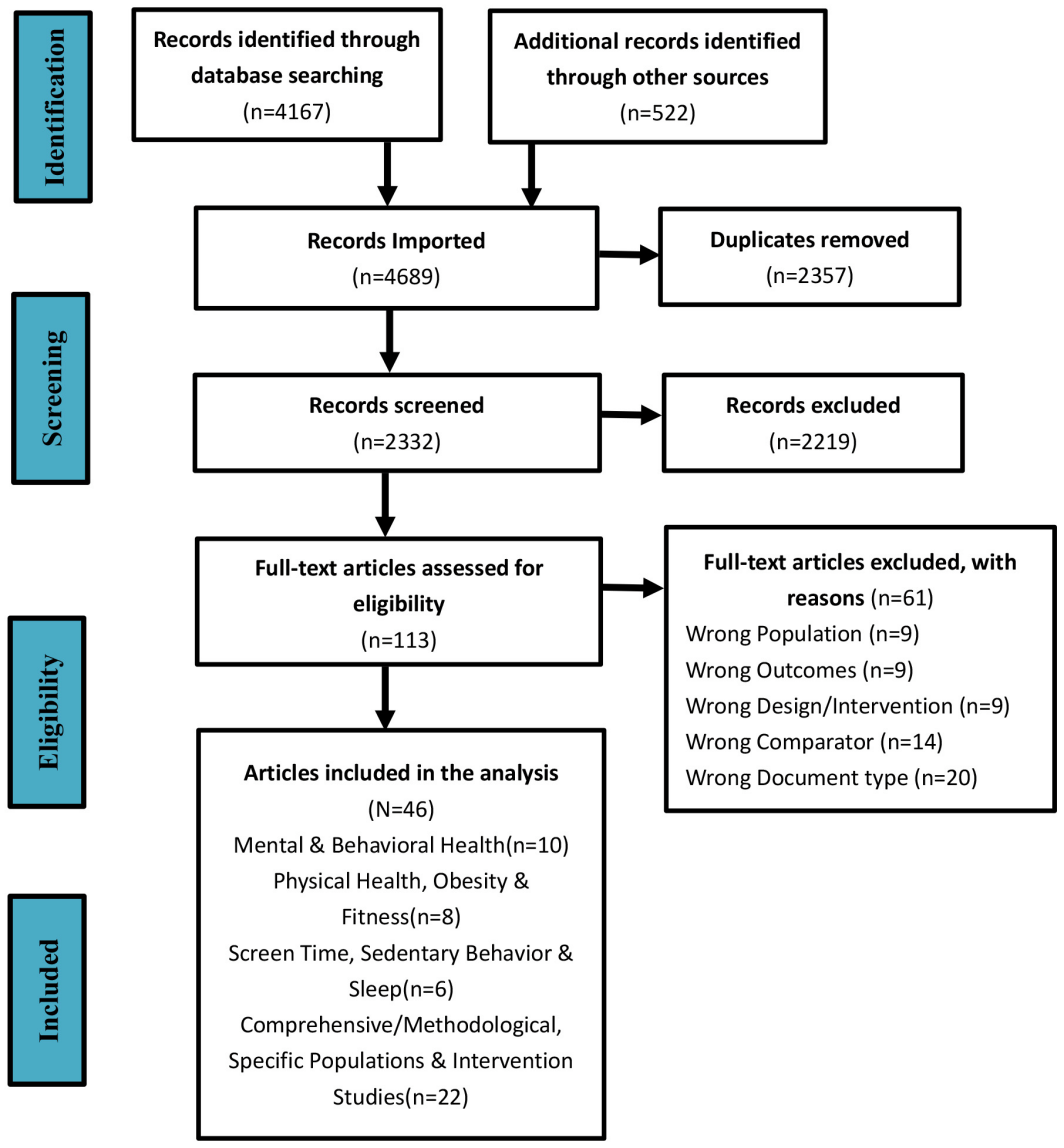
PRISMA-style flow diagram illustrating the literature search and screening process. This adapted flowchart is used to enhance transparency and does not imply that this narrative review follows the full PRISMA systematic review protocol.

In the initial search, 4,684 articles that could be relevant were found. Following the removal of duplicates, 108 full-text papers were assessed for potential inclusion after examining their titles and abstracts. In the end, 46 studies fulfilled the eligibility requirements and were incorporated into the narrative review. Figure 1 presents a flow diagram following the PRISMA-style format, which shows the quantity of studies eliminated at each stage of the narrative synthesis, highlighting the search and screening methodology employed in this narrative review.

### Thematic classification of included studies

The analysis encompassed 46 studies, which were categorized into four primary themes based on their focal points: mental and behavioral health (10 studies), Physical Health, Obesity, and Fitness (8 studies), Screen Time, Sedentary Behavior, and Sleep (6 studies), and Comprehensive/Methodological, Specific Populations, and Intervention Studies (22 studies). A thorough classification can be found in Table 1, detailing reference numbers and the main emphasis of each category. The subsequent Results section outlines the significant findings, structured around these four thematic areas: outcomes related to mental health; results concerning physical health, obesity, and fitness; aspects of sedentary behavior, screen time, and sleep; as well as gradients of activity intensity and evidence from interventions.

**Table 1:** Thematic classification of included articles.

Category	Number of articles	Reference numbers	Key focus
Mental & behavioral health	10	6, 7, 10, 11, 17, 27, 31, 34, 35, 45	Relationship between physical activity, sedentary behavior, and psychological indicators (depression, anxiety, self-esteem, life satisfaction, subjective health).
Physical health, obesity, and fitness outcomes	8	3, 5, 19, 22, 23, 29, 30, 36, 44	Effects of physical activity on obesity, body composition, fitness, chronic disease management (e.g., diabetes), and specific physical pain (e.g., back, neck pain).
Sedentary behavior, screen time, & sleep	6	2, 4, 21, 25, 38, 39	Screen time, sedentary behavior, sleep as core variables; their substitution/association with physical activity and impact on comprehensive health (especially mental health).
Activity intensity gradients and intervention evidence	22	1, 8, 9, 12, 13, 14, 15, 16, 18, 20, 24, 26, 28, 32, 33, 37, 40, 41, 42, 43, 44, 45	Includes systematic reviews/meta-analyses (7, 15, 17, 38–40), intervention studies & technology/methods research (16, 18, 28, 33, 36, 37, 41), and studies on special populations, health equity & promotion strategies (2, 6, 34, 42–45).

In Table 1, a thematic overview of the 46 included studies is presented, divided into four primary categories. The Results section outlines the findings based on these categories. For readers seeking comprehensive details on each study, Appendix A1–4 provides categorized tables by theme, including reference details and the aims of each research.

### Mental health outcomes associated with physical activity

A total of ten investigations focusing on mental health results (13, 14, 20–27), along with findings from systematic reviews (17) and intervention studies (28), contribute to the following synthesis. The compiled evidence consistently indicates a favorable link between physical activity and the mental wellbeing of adolescents (13, 14, 17, 21, 22, 24). Several studies have shown that engaging in moderate to vigorous physical activity (MVPA) correlates with lower levels of depression and anxiety, as well as improved self-esteem and overall life satisfaction (13, 14, 17, 21, 22, 24). In a cluster randomized controlled trial conducted by Åvitsland et al., it was revealed that a school-based physical activity initiative resulted in a 22% decrease in depression scores among participants who initially exhibited the most severe psychological symptoms, surpassing the results observed in the control group (28).

The connection between exercise and mental wellbeing seems to be influenced by various psychosocial elements. Research by An et al. revealed that self-esteem and symptoms of depression play crucial roles in linking physical activity to thoughts of suicide. Likewise, Li et al. found that the frequency of physical activity affects overall mental health through life satisfaction and self-assessed health, with varying impacts depending on how often one exercises: occasional workouts mainly improve life satisfaction, whereas regular exercise is more effective in enhancing self-rated health. These mediating factors are further corroborated by findings from systematic reviews that explore psychosocial mechanisms.

Numerous studies have highlighted gender disparities in these relationships (20, 21, 25, 26). According to Halliday et al., adolescent females tend to have worse mental health and participate in less physical activity than their male counterparts, indicating that physical activity might help clarify the gender differences observed in adolescent mental health (21). Moreover, the environment in which physical activity occurs is significant: Werneck et al. discovered that physical activity related to commuting correlated with increased chances of anxiety-related sleep issues, while physical activity in schools appeared to offer protection against these problems (23). Further insights into the effects of context are provided by research that investigates various physical activity environments and their unique influences on mental health (22, 23, 26).

To conclude, research consistently indicates a beneficial relationship between physical exercise and the mental wellbeing of adolescents, especially regarding moderate to vigorous physical activity (MVPA), which is associated with lower levels of anxiety and depression, as well as improved self-esteem and overall life satisfaction. Nonetheless, the strength of these effects differs based on factors such as gender, the context of the activity (like commuting compared to school-related activities), and how often the activity occurs, pointing to significant variables that require further exploration. Additionally, the influence of self-esteem, symptoms of depression, life satisfaction, and self-assessed health underscores the psychological mechanisms at play.

### Physical health, obesity, and fitness outcomes

This section compiles findings from eight studies that examine physical health outcomes (4, 5, 11, 12, 29–32) alongside insights on activity intensity levels (6, 7), the effects of interventions (33), and considerations for specific populations (19) to create a thorough summary. The data strongly indicate that engaging in physical activity is beneficial for the physical health of adolescents (4, 5, 7, 29–31). Research by García-Hermoso et al. identified a notable negative relationship between vigorous physical activity (VPA) and total body fat (*r* = −0.09–−0.13), as well as a positive association between VPA and cardiorespiratory fitness (CRF) (*r* = 0.25) (7). These results align with earlier studies that focused on body composition and fitness metrics (4, 5, 27, 29–31). Additionally, intervention research shows that enhancing daily physical activity can significantly decrease body fat percentage (5, 33).

Significantly, even low-intensity physical activity (LPA) appears to confer health benefits (2, 6, 7, 9). Dahlstrand et al. found that increases in LPA were associated with favorable changes in certain cardiovascular health metrics, whereas sedentary behavior (SB) and declines in LPA were linked to elevated BMI z-scores, waist circumference, and resting heart rate (6). This suggests that any movement toward a more active lifestyle across the intensity spectrum may yield health benefits, a conclusion supported by research on activity intensity gradients (2, 6, 7).

The timing of physical activity may also influence health outcomes (30). Stavinski et al. reported that afternoon MVPA was associated with better performance in fitness assessments (including the 20-meter shuttle run and standing long jump), while morning MVPA correlated with healthier BMI but showed a negative relationship with 40-meter sprint performance (30). This emerging area of research suggests potential interactions between circadian rhythms and exercise effects on adolescent health.

Physical activity also plays a crucial role in managing chronic conditions (5, 19, 31, 33, 34, 34). Among adolescents with type 1 diabetes, Aljawarneh et al. found significant associations between physical activity participation, health-related quality of life, regimen adherence, and glycemic control (31). Similarly, for adolescents with attention deficit hyperactivity disorder (ADHD), Liang et al. reported a notable link between higher amounts of MVPA and enhanced executive functions (*k* = 9, *r* = 0.36, 95% CI 0.22–0.49), as well as a negative correlation between MVPA and mental health issues (*k* = 5, *r* = −0.19, 95% CI−0.35–−0.03) (19). Additional evidence on physical activity benefits for special populations is provided by studies examining overweight adolescents (5, 33) and those with chronic conditions (31, 34, 34).

The available research suggests that engaging in physical activity at various intensity levels offers numerous health advantages, such as enhanced body composition, improved cardiorespiratory fitness, and more effective control of chronic illnesses. Interestingly, even activities of low intensity provide significant cardiovascular benefits, although the timing of these activities can lead to varying results. The reliability of these findings across different demographic groups highlights the strength of these connections, yet the relationship between activity levels and outcomes, as well as the effects of timing, need additional investigation.

### Sedentary behavior, screen time, and sleep

A collection of six studies focusing on sedentary behavior and screen usage (3, 8–10, 19, 35), along with supplementary data regarding musculoskeletal issues (11, 12) and protective elements (20), underpins the following analysis. Sedentary lifestyles and high screen time are identified as separate risk factors impacting adolescent wellbeing, differing from the consequences of inadequate physical activity (3, 8–12, 19, 32, 35). Extended screen exposure (over 3 h daily) correlates with heightened risks of obesity, as well as discomfort in the lower back and neck/shoulder areas (10–12, 32, 35). Research by Myrtveit et al. indicated that frequent interactive screen engagement (such as gaming and social networking) leads to moderate increases in neck and shoulder pain (12), while Bento et al. found significant links between excessive screen time and low back pain, especially when accompanied by lying or semi-reclined positions (11). These results align with studies exploring the connection between screen-related activities and physical ailments (9, 32).

Research conducted by Nygaard and colleagues through latent class analysis indicated that adolescents who displayed a combination of \high screen time and low sleep\ experienced the worst mental health effects, such as heightened physical complaints and poor body image (10). This aggregation of harmful behaviors highlights the increased dangers linked to contemporary sedentary behavior, a conclusion backed by investigations into the relationship between screen usage, sleep patterns, and mental wellbeing (3, 8, 19, 35).

Crucially, engaging in physical exercise can mitigate several of these detrimental impacts (6, 20, 35). Research by Farren et al. revealed that elevated cardiorespiratory fitness levels, as measured by VOmax, along with sufficient participation in moderate to vigorous physical activity (MVPA), can reduce the negative consequences of extended sitting on symptoms of depression (20). This discovery holds significant implications for public health: in a society dominated by screens, encouraging MVPA could serve as a protective \immune barrier\ for the mental wellbeing of adolescents, as indicated by studies examining the interplay between physical activity and sedentary lifestyles.

Table 2 offers an in-depth analysis of the unique hazards associated with different sedentary activities and screen time, highlighting their impacts on physical wellbeing, mental health, and musculoskeletal pain (6, 20, 35).

**Table 2:** Multidimensional health risk assessment of sedentary and screen-based behaviors.

Behavior category	Risk to physical health	Risk to mental health	Association with musculoskeletal pain	Key evidence/behavioral context
General sedentary behavior	High (obesity, metabolic risk)	Moderate (depressive symptoms)	Potential risk	Associated with higher BMI, waist circumference, resting heart rate
Prolonged screen time (>3 h/day)	Moderate-high (obesity, low back pain)	Moderate-high (depression, poor body image)	Strong (especially low back pain)	Lying/semi-lying posture, prolonged TV viewing
High-frequency interactive screen use (e.g., gaming, social media)	Moderate (neck/shoulder pain)	Moderate (anxiety, stress)	Moderate (neck/shoulder pain)	Slightly increased risk of neck/shoulder pain
Screen time with short sleep	Compounded risk	High (psychological & somatic symptoms)	Insufficient data	Cluster analysis shows poorest mental health profile for this pattern

Risk level (high, moderate) is a synthetic assessment based on the strength and consistency of reported associations in the cited literature. These categorizations are intended as narrative, interpretive overviews to facilitate understanding of research trends, rather than formal evidence grading or quantitative risk prediction.

To conclude, sedentary behavior, especially related to screen usage, significantly heightens the likelihood of obesity, bodily discomfort, and mental health issues, with these risks intensified when combined with inadequate sleep. Engaging in physical activity can mitigate some of these adverse effects, emphasizing the relationship between various movement patterns. The varying impacts observed across different screen activities and demographic groups point to the necessity for tailored intervention strategies.

### Activity intensity gradients and intervention evidence

Multiple studies have examined how different intensities of physical activity—from sedentary behavior to vigorous activity—differentially influence health outcomes (2, 6, 7, 35). The relationship between physical activity and health outcomes follows a clear intensity gradient (2, 6, 7, 35). As intensity increases from sedentary behavior to vigorous physical activity, there is a progressive enhancement in cardiorespiratory fitness and bone density outcomes (7, 35). VPA shows the strongest correlations with improvements in these areas (7, 30), while MVPA appears particularly important for body composition and mental health (6, 13, 14, 17). Notably, LPA demonstrates health benefits distinct from higher-intensity activities, making it a practical target for adolescents with limited capacity or motivation (2, 6). Research examining the full spectrum of activity intensities consistently supports this graded relationship (2, 6, 7, 35). Table 3 summarizes the relative effects of each intensity category on key physical and mental health indicators.

**Table 3:** Relative effects of different PA intensities on key health indicators in adolescents.

Health indicator	Sedentary behavior (SB)	Light PA (LPA)	Moderate-to-vigorous PA (MVPA)	Vigorous PA (VPA)	Key supporting references
Body fat/BMI z-score	Positive association (↑)	Weak or neutral	Negative association (↓)	Negative association (↓)	(6, 7)
Cardiorespiratory fitness	Negative association (↓)	Positive, but weak	Positive association (↑)	Strong positive association (↑)	(7)
Depressive/anxiety symptoms	Positive association (↑)	Negative association (↓)	Negative association (↓)	Negative association (↓)	(2, 6, 17)
Self-Esteem	Negative association (↓)	Positive association (↑)	Positive association (↑)	Positive association (↑)	(13, 17)
Bone Mineral Density	Weak/Neutral	Weak/Neutral	Positive association (↑)	Positive association (↑)	(7)

Arrows (↑/↓) indicate the direction of association between the behavior and the health indicator. Bold text highlights the intensity category with the strongest relative benefit or most consistent evidence for that specific indicator. These judgments are based on the authors' synthesis of included literature and are intended to illustrate research trends rather than provide precise effect size estimates.

Leveraging the concept of intensity gradients, research involving interventions demonstrates a direct link between physical activity and health advantages, while also providing actionable guidance on how to attain these benefits in various environments and among diverse groups.

A selection of the studies reviewed focused on the impact of physical activity initiatives, which encompassed programs implemented in schools (28), digital health applications (16, 18, 33), culturally specific strategies (5), and mobile interventions that incorporate gaming elements (36). These intervention studies offer evidence of a causal relationship between physical activity and health benefits (5, 15, 18, 28, 29, 33, 36–38). Notably, school-based programs have shown significant potential (28, 29, 37). For instance, the \School in Motion\ cluster randomized controlled trial conducted in Norway revealed that a comprehensive physical activity initiative resulted in marked improvements in mental health, especially for students experiencing high levels of psychological distress at the outset (28). Additionally, other school-based programs have reported comparable positive outcomes in terms of physical fitness and academic achievement (29, 37).

Digital health strategies are becoming a novel method for encouraging physical activity in adolescents (16, 18, 33, 36). A systematic review by Böhm et al. examined mobile health solutions, such as wearable fitness trackers, and found moderate support for their role in enhancing physical activity among healthy youth (18). Cummings et al. highlighted favorable outcomes from a digital health initiative aimed at increasing activity levels in overweight or obese adolescents, noting enhancements in both physical activity and body composition (33). Further studies on technology-driven interventions have investigated elements like gamification, mobile health apps (16), and digital behavior modification techniques (16, 18, 33).

Interventions customized to cultural contexts can increase their effectiveness (5, 37, 38). Srivastav and colleagues found that a well-organized fitness initiative, which included educational assistance and behavioral coaching, notably enhanced the health-related quality of life for overweight adolescents in India, positively affecting their physical, emotional, social, and academic wellbeing (5). Likewise, health education strategies focused on skill development have proven effective in encouraging physical activity among various adolescent groups (37). Studies on designing interventions consistently highlight the necessity of adapting to the unique characteristics of specific populations and settings (5, 15, 28, 36–38).

To sum up, there is a clear connection between the intensity of physical activity and health results, with vigorous physical activity (VPA) having the most significant impact on cardiorespiratory fitness, moderate to vigorous physical activity (MVPA) benefiting mental health and body composition, and light physical activity (LPA) providing easily attainable advantages. Research on interventions supports these conclusions, showing that programs designed for schools, digital platforms, and specific cultural contexts can successfully encourage physical activity. Nonetheless, the diversity in intervention strategies and target demographics highlights the need for uniform assessment methods and tailored approaches.

## Discussion

### The multidimensional nature of the activity-health relationship

This narrative review synthesizes evidence demonstrating that the relationship between physical activity and adolescent health is not uniform but operates through a complex regulatory network involving intensity, type, context, and individual characteristics (2, 6, 7, 9, 35). The findings consistently support a dose-response relationship wherein higher intensity activity yields greater benefits for cardiorespiratory fitness and bone health, while moderate intensity activity appears optimal for mental health outcomes (7, 13, 14, 17). Importantly, the evidence also reveals that even light-intensity activity confers measurable health benefits, challenging the traditional focus on moderate-to-vigorous activity alone and supporting a more inclusive, ”movement-oriented‘' approach to health promotion (2, 6, 9).

### Bridging physiological and psychosocial pathways

The mechanisms linking physical activity to adolescent health outcomes operate through multiple interconnected pathways (6, 7, 13, 14, 17, 39). Physiologically, moderate to vigorous physical activity facilitates negative energy balance through increased energy expenditure, leading to reductions in body fat (7, 22). Both MVPA and VPA enhance heart rate regulation and vascular health, whereas sedentary behavior and LPA correlate with unfavorable metabolic indicators such as elevated diastolic blood pressure and increased pulse wave velocity (6). Neuroendocrine mechanisms, including endorphin release and brain-derived neurotrophic factor elevation, likely contribute to the mental health benefits observed, although direct evidence in adolescent populations remains limited (17, 39).

Psychosocially, physical activity influences mental health through enhanced self-esteem, mastery experiences, and social connectedness (13, 14, 17, 39). The mediating roles of self-esteem and depressive symptoms in the relationship between physical activity and suicidal ideation suggest that activity-based interventions may be particularly valuable when combined with strategies targeting these psychological constructs (13). Similarly, the differential mediating effects of life satisfaction and self-rated health depending on activity frequency point to distinct psychological pathways that may be activated at different activity levels (14). These psychosocial mechanisms are further supported by systematic reviews examining the pathways linking physical activity to mental wellbeing (17, 39).

Future research directions

This review identifies several priority areas for future research. First, well-designed longitudinal studies and mechanistic clinical trials are needed to establish causal relationships and elucidate the timing and dose-response characteristics of activity-health associations. Second, research should move beyond simplistic ”activity volume‘' metrics to examine the specific effects of activity type, context, and timing, including the emerging area of circadian influences on exercise responses. Third, cross-cultural comparative studies are essential to distinguish universal principles from culturally specific patterns and to inform the development of culturally sensitive interventions. Fourth, the application of advanced analytical methods, including isotemporal substitution analysis and compositional data analysis, can address the interdependent nature of movement behaviors within the 24-h cycle. Fifth, research should prioritize understudied populations, including adolescents with chronic health conditions, disabilities, and those in marginalized communities, to ensure that the evidence base supports equitable health promotion.

### Gender, culture, and socioeconomic factors

The observed heterogeneity in activity-health associations across studies reflects meaningful moderating effects that warrant attention (20, 21, 25, 26, 34, 38). Consistent gender differences emerge, with girls consistently reporting poorer mental health outcomes and lower activity participation (20, 21, 25, 26). These disparities likely reflect a complex interplay of biological, psychological, and sociocultural factors, including differential socialization regarding physical activity, body image concerns, and access to opportunities (21, 25, 26).

Cultural and socioeconomic contexts similarly shape both activity patterns and their health implications (5, 34, 37, 38). The higher obesity prevalence and lower MVPA engagement among certain population subgroups illustrate how racial/ethnic disparities in health outcomes may be partially attributable to differential activity patterns, which in turn reflect broader structural inequalities in access to safe recreational spaces, quality physical education, and culturally relevant activity options (5, 33, 34, 38). Research on culturally tailored interventions demonstrates that addressing these contextual factors can enhance intervention effectiveness (5, 37, 38).

### Methodological challenges and implications for evidence interpretation

Several methodological issues limit the strength of conclusions that can be drawn from the current evidence base (2, 15–18, 40, 41). First, the predominance of cross-sectional designs precludes causal inference, leaving open the possibility of reverse causation or unmeasured confounding (15, 17, 25). Second, measurement challenges persist: while accelerometers provide objective activity assessment, they fail to capture non-ambulatory activities such as cycling, swimming, and resistance training, potentially underestimating total activity volume and misclassifying intensity (2, 6, 18, 41). Third, the field lacks standardized definitions for activity types, intensity thresholds, and sedentary behavior, hampering cross-study comparability (2, 7, 40). Fourth, most studies examine behaviors in isolation rather than adopting a 24-h movement framework that accounts for the compositional nature of time use and the interdependent relationships between sleep, sedentary behavior, LPA, and MVPA (3, 8, 35).

### Implications for intervention design and health promotion

The evidence synthesized in this review carries several implications for intervention design (5, 15, 18, 28, 33, 36–38). First, the dose-response gradient across activity intensities suggests that interventions should adopt a ”something is better than nothing‘' philosophy while still encouraging progression toward higher intensities when feasible (2, 6, 9). Replacing sedentary time with light activity may be a more achievable initial target for inactive adolescents than aiming directly for MVPA.

Second, the moderating effects of gender, culture, and socioeconomic context underscore the need for tailored rather than one-size-fits-all approaches (5, 21, 25, 26, 34, 37, 38). Interventions that are culturally relevant, address gender-specific barriers, and account for socioeconomic constraints on activity opportunities are more likely to achieve meaningful and sustained behavior change (5, 37, 38).

Third, the clustering of risky behaviors (e.g., excessive screen time with insufficient sleep) highlights the importance of multicomponent interventions that address multiple behaviors simultaneously (3, 8, 10, 19, 35). School-based interventions that integrate physical activity promotion with sleep hygiene education and screen time reduction strategies may prove more effective than single-behavior approaches (3, 8, 10, 28).

Fourth, the potential of digital health technologies to reach adolescents in their natural environments warrants further exploration (16, 18, 33, 36). Mobile health interventions incorporating gamification, personalization, and social support elements have shown promise, but questions remain regarding optimal design features, long-term engagement, and effectiveness across diverse populations.

### Future research directions

This analysis highlights several key areas that warrant further investigation (2, 3, 6–8, 14, 15, 17, 40, 41). Firstly, there is a need for well-structured longitudinal studies and mechanistic clinical trials to clarify causal links and explore the timing and dose-response relationships associated with activity and health (14, 15, 17, 25). Secondly, research should advance beyond basic “activity volume” measures to investigate the distinct impacts of activity type, context, and timing, particularly the emerging field of circadian effects on exercise responses (2, 6, 30, 41). Thirdly, cross-cultural comparative research is crucial for differentiating universal principles from culturally specific trends, aiding in the creation of culturally appropriate interventions (5, 21, 34, 37, 38). Fourthly, utilizing sophisticated analytical techniques, such as isotemporal substitution analysis and compositional data analysis, can help address the interconnected nature of movement behaviors throughout the 24-h period (3, 8, 35). Fifthly, research efforts should focus on underrepresented groups, including adolescents with chronic health issues, disabilities, and individuals from marginalized communities, to ensure that the evidence base promotes equitable health initiatives (19, 31, 34, 38). Lastly, future intervention studies should adopt robust designs with extended follow-up durations and investigate mediators and moderators of intervention outcomes to pinpoint effective components and enhance intervention strategies (5, 15, 18, 28, 33, 36, 37).

### Limitations

This narrative review has several limitations that should be acknowledged (15, 17, 18, 40). First, the purposive sampling of literature, while appropriate for a narrative review, may have introduced selection bias and does not provide the exhaustive coverage characteristic of systematic reviews (17, 40). Second, the exclusion of non-English language publications may have omitted relevant evidence from non-English speaking contexts (15, 17). Third, the heterogeneity of included studies in terms of design, measurement, and outcome definitions precluded quantitative synthesis and limited the precision of conclusions (7, 15, 17, 40). Fourth, the narrative synthesis approach, while allowing for interpretive flexibility, may be subject to author bias in the selection and interpretation of evidence (17, 18). Fifth, the review's focus on general adolescent populations may limit applicability to specific subgroups with unique needs or circumstances (19, 31, 34, 38). Sixth, while the study sample includes participants from diverse cultural and socioeconomic backgrounds, its findings cannot fully account for how cultural norms, value systems, and contextual factors moderate the associations between physical activity, sedentary behavior, and health outcomes across population subgroups (5, 21, 34, 37, 38). Seventh, the lack of formal quality assessment or sensitivity analysis, while consistent with narrative review methodology, means that study quality was not systematically incorporated into evidence synthesis (15, 17, 40).

## Conclusion

This review offers an in-depth analysis and thoughtful examination of the intricate connections between physical activity, inactivity, and the health of adolescents. By consolidating and assessing existing research, it goes further than merely outlining fundamental correlations to investigate the deeper patterns, mechanisms, contextual differences, and methodological issues involved.

This review compiles evidence indicating that the health of adolescents is influenced by a range of movement behaviors instead of relying on a single form of activity. Engaging in moderate to vigorous physical activity is linked to better physical health results, such as improved body composition and enhanced cardiorespiratory fitness, along with positive mental health effects, including lower levels of depression and anxiety and increased self-esteem. Notably, the review emphasizes the complex influences of intensity, type, timing, and context on health outcomes: even light physical activity can provide cardiovascular advantages, while more intense activities are more strongly correlated with stress alleviation. Additionally, the environment where the activity takes place—be it in schools, during commutes, or in recreational settings—affects its impact on mental health.

Inactivity, especially when it involves prolonged screen usage, stands out as a distinct threat to health. Extended periods of being sedentary are linked to weight gain, metabolic issues, physical discomfort, and negative effects on mental wellbeing. Notably, these habits frequently occur together; the pairing of high screen time with inadequate sleep results in the worst mental health conditions, highlighting the importance of comprehensive intervention strategies.

The connections between physical exercise and mental wellbeing are becoming clearer. Factors such as self-worth, symptoms of depression, overall life satisfaction, and personal health assessments play crucial roles in bridging the gap between engaging in physical activity and mental health results, including thoughts of suicide. The nature of these mediating factors may vary based on how often individuals participate in physical activities, which can inform the development of specific intervention strategies.

This analysis highlights major methodological shortcomings in the existing body of evidence that hinder causal conclusions and broader applicability. These issues encompass difficulties in measurement, limitations in study design, a lack of focus on diverse populations, and inconsistencies in definitions. In light of this evaluation, the review suggests a detailed framework for upcoming research and practice, prioritizing tailored methods, causal analysis, cross-cultural viewpoints, and cohesive intervention strategies.

This narrative review highlights the intricate nature, advancements, and ongoing difficulties in the realm of adolescent health and physical activity. Attaining the best health results for young people necessitates more than generic solutions; it demands a thoughtful, context-aware, and comprehensive approach based on the most reliable evidence. Strengthening the links between research, practice, and policy is essential. By persistently improving our methods, investigating fundamental processes, and creating customized strategies, we can better promote the long-term physical and mental health of adolescents while tackling the increasing issues related to chronic illnesses and mental health challenges.
