# Supplementary material for: Physical activity, sedentary behavior, and adolescent health: a narrative review
Source: Front Public Health. 2026 Apr 23;14:1809745. doi: 10.3389/fpubh.2026.1809745 (PMC13149281; doi:10.3389/fpubh.2026.1809745)
Supplement: Supplementary file 1 [file Supplementary_file_1.docx]

**Appendix A1-4: Detailed characteristics of included studies**

This appendix contains tables that offer comprehensive details about the 46 studies featured in this narrative review. The studies are categorized according to the four themes outlined in Table 1, aligning with the sections found in the Results. This structure enables readers to follow the studies from their respective categories to the synthesis of thematic insights.

**Table A1.** Mental and behavioral health outcomes of physical activity in adolescents

| Category | Reference Numbers | First Author | Year | Research Focus (Core Content) |
| --- | --- | --- | --- | --- |
| Mental & Behavioral Health | 6 | An D | 2024 | Examines the associations between physical activity and depression, self-esteem, and suicidal ideation. |
|  | 7 | Li W | 2024 | Re-examines the impact of physical activity frequency on mental health, life satisfaction, and self-rated health. |
|  | 10 | Farren G L | 2017 | Investigates the role of sedentary behavior and physical activity in predicting depressive symptoms, independent of fitness attributes. |
|  | 11 | Halliday A J | 2019 | Explores whether physical activity can help explain the gender gap in adolescent mental health. |
|  | 17 | Chi X | 2021 | Studies the relationship between mental health problems, nutrition, and physical activity among Chinese adolescents during COVID-19. |
|  | 27 | Werneck A O | 2020 | Analyzes the association between different contexts of physical activity and anxiety-induced sleep disturbance. |
|  | 31 | Vedøy I B | 2020 | Investigates the cross-sectional relationships between physical activity, mental health, and academic achievement. |
|  | 34 | Herman K M | 2015 | Explores the relationships between physical activity, screen time, and self-rated health/mental health. |
|  | 35 | Halladay J | 2024 | Explores the relationship between sports participation, physical activity, belongingness, and patterns of substance use and mental health. |
|  | 45 | Roren A | 2023 | Provides a critical narrative review of the relationship between physical activity and low back pain, highlighting preventive and therapeutic effects |

**Table A2.** Physical health, obesity, and fitness profiles associated with physical activity in adolescents

| Category | Reference Numbers | First Author | Year | Research Focus (Core Content) |
| --- | --- | --- | --- | --- |
| Physical Health, Obesity & Physical Fitness | 3 | Li H | 2025 | Investigates the association between physical activity, smartphone usage, and obesity risk among Korean adolescents. |
|  | 5 | Srivastav P | 2025 | Evaluates the impact of physical activity and BMI on health-related quality of life in overweight Indian adolescents. |
|  | 19 | Kang P J | 2024 | Investigates the effects of physical activity on fitness, working memory, and academic performance. |
|  | 22 | Bento T P F | 2020 | Investigates sociodemographic associations of low back pain with electronic device use, physical activity, and mental health in adolescents. |
|  | 23 | Myrtveit S M | 2014 | Examines the association of adolescent neck/shoulder pain with depression, physical activity, screen-based activities, and healthcare use. |
|  | 29 | Stavinski N | 2025 | Analyzes associations between the timing of device-measured physical activity and health-related physical fitness indicators. |
|  | 30 | Aljawarneh Y M | 2023 | Examines associations between physical activity, health-related quality of life, regimen adherence, and glycemic control in adolescents with type 1 diabetes. |
|  | 36 | Nilsen S A | 2023 | Analyzes trends in physical health complaints among adolescents (2014–2019), considering screen time, social media use, and physical activity. |

**Table A3.** Interplay of physical activity, screen time, sedentary behavior, and sleep in adolescent health

| Category | Reference Numbers | First Author | Year | Research Focus (Core Content) |
| --- | --- | --- | --- | --- |
| Screen/  Sedentary/  Sleep | 2 | Gilchrist J D | 2021 | Investigates the effects of reallocating time between physical activity, sedentary behaviors, and sleep on mental health. |
|  | 4 | Liang X | 2025 | A systematic review and meta-analysis of associations between accelerometer-measured physical activity, sleep duration, and health indicators in children/adolescents with ADHD. |
|  | 21 | Nygaard M | 2025 | Uses latent class analysis to identify movement patterns (screen use, PA, sleep) in Danish adolescents and correlates them with mental health. |
|  | 25 | Duncan M J | 2022 | A longitudinal isotemporal substitution analysis of the association between PA, sleep, screen time, and mental health in Canadian adolescents during COVID-19. |
|  | 38 | Tebar W R | 2021 | (See above) Highlights the independent negative effect of sedentary behavior on self-rated health. |
|  | 39 | Clayborne ZM | 2025 | Investigates associations between digital media use behaviours, screen time, and positive mental health in Canadian youth. |

**Table A4.** Methodological reviews, targeted interventions, and population-specific studies in adolescent physical activity research

| Category | Reference Numbers | First Author | Year | Research Focus (Core Content) |
| --- | --- | --- | --- | --- |
| Systematic Reviews & Meta-Analyses | 12 | Biddle S J | 2018 | An updated umbrella review on physical activity and mental health in children/adolescents, including a causality analysis. |
|  | 14 | García-Hermoso A | 2021 | A systematic review and meta-analysis on the association between device-measured vigorous PA and health outcomes in children/adolescents. |
|  | 26 | Dzhambov A M | 2023 | A scoping review on the protective effect of restorative possibilities (including PA) on cognitive function and mental health. |
|  | 28 | Neill R D | 2020 | A systematic review and meta-analysis on the effects of interventions with PA components on adolescent mental health. |
|  | 33 | Sakhvidi M J Z | 2022 | An overview of systematic reviews on greenspace and health, wellbeing, PA, and development in children/adolescents. |
|  | 41 | Wilson B | 2020 | A systematic review of PA interventions aimed at improving the health of children/adolescents in out-of-home care. |
|  | 44 | Milton K | 2024 | Examines global and national physical activity and sedentary behaviour guidelines development processes, addressing methodological standards |
| Intervention Studies, Technology & Methodology | 13 | Böhm B | 2019 | A systematic review on the effects of mobile health (including wearables) to increase PA outcomes among healthy children/adolescents. |
|  | 15 | Cummings C | 2022 | An open trial of a digital health program targeting PA among adolescents with overweight/obesity. |
|  | 20 | Rahayu A | 2026 | Evaluates the promotion of PA among adolescents through skill-based health education. |
|  | 24 | Åvitsland A | 2020 | Reports results from the "School in Motion" cluster RCT, assessing the effects of a school-based PA intervention on mental health. |
|  | 32 | Lee A M | 2019 | A scoping review on the efficacy and effectiveness of mobile health technologies for facilitating PA in adolescents. |
|  | 37 | Larouche R | 2024 | Study protocol for the development and validation of the Global Adolescent and Child Physical Activity Questionnaire (GAC-PAQ). Interventions & Technology (Measurement Tool) |
|  | 40 | Pyky R | 2017 | A population-based RCT evaluating a tailored, gamified mobile PA intervention on life satisfaction and self-rated health in adolescent males. |
| Specific Populations, Health Equity & Promotion Strategies | 1 | D'Agostino E M | 2023 | A commentary exploring new frontiers in PA intensity measurement and its association with adolescent health. |
|  | 8 | Dahlstrand J | 2023 | (Inferred from title) Discusses providing precision in adolescent health and PA behavior patterns. |
|  | 9 | Silva A O da | 2018 | Investigates the association between health self-perception, PA, and nutritional status in adolescents. |
|  | 16 | Talotta R | 2024 | Narrative review on the mental health benefits (e.g., on depression, anxiety) and potential mechanisms of physical activity for patients with fibromyalgia. Specific Populations & Strategies. |
|  | 18 | Costa B G G da | 2020 | Examines the association between practicing sport/non-sport PAs and health-related quality of life in Brazilian adolescents. |
|  | 42 | Casey M | 2016 | Analyzes patterns of time use and their associations with PA correlates and quality of life among regional/rural adolescent girls. |
|  | 43 | Bush P L | 2016 | A case study of a researcher-organization partnership to optimize adolescent health and PA programming at a YMCA. Specific Populations & Strategies. |
|  | 46 | Talotta R | 2024 | Explores mental health benefits and potential mechanisms of physical activity for patients with fibromyalgia. |
